# Metabolic Adaptation in Transplastomic Plants Massively Accumulating Recombinant Proteins

**DOI:** 10.1371/journal.pone.0025289

**Published:** 2011-09-22

**Authors:** Julia Bally, Claudette Job, Maya Belghazi, Dominique Job

**Affiliations:** 1 Centre National de la Recherche Scientifique - Bayer CropScience Joint Laboratory, UMR5240, Lyon, France; 2 Centre d'Analyse Protéomique de Marseille, Institut Fédératif de Recherche Jean Roche, Marseille, France; University of South Florida College of Medicine, United States of America

## Abstract

**Background:**

Recombinant chloroplasts are endowed with an astonishing capacity to accumulate foreign proteins. However, knowledge about the impact on resident proteins of such high levels of recombinant protein accumulation is lacking.

**Methodology/Principal Findings:**

Here we used proteomics to characterize tobacco (*Nicotiana tabacum*) plastid transformants massively accumulating a *p*-hydroxyphenyl pyruvate dioxygenase (HPPD) or a green fluorescent protein (GFP). While under the conditions used no obvious modifications in plant phenotype could be observed, these proteins accumulated to even higher levels than ribulose 1,5-bisphosphate carboxylase/oxygenase (Rubisco), the most abundant protein on the planet. This accumulation occurred at the expense of a limited number of leaf proteins including Rubisco. In particular, enzymes involved in CO_2_ metabolism such as nuclear-encoded plastidial Calvin cycle enzymes and mitochondrial glycine decarboxylase were found to adjust their accumulation level to these novel physiological conditions.

**Conclusions/Significance:**

The results document how protein synthetic capacity is limited in plant cells. They may provide new avenues to evaluate possible bottlenecks in recombinant protein technology and to maintain plant fitness in future studies aiming at producing recombinant proteins of interest through chloroplast transformation.

## Introduction

The genetic modification of the plastid genome recently emerged as an alternative to nuclear transformation in fundamental research, for example to understand the interactions between the chloroplast and nuclear genomes, but also in applied research, as a system of transgene expression for high-added-value protein production. Chloroplast transformation [1]–[2] allows extremely high accumulation of recombinant proteins e.g., up to 70% of total soluble proteins [3], a feature that is of utmost importance for crop improvement and molecular farming. Very little attention has been paid however toward understanding the mechanisms accounting for such capacity of chloroplasts to massively accumulate recombinant proteins. Previously, we addressed this question by engineering tobacco plants through plastid transformation that expressed recombinant proteins of different nature and origin [4]. While under the conditions used no obvious modifications in plant phenotype could be observed, the accumulation level of the ribulose 1,5-bisphosphate carboxylase/oxygenase (Rubisco) subunits, the most abundant protein complex in leaves, strongly dropped in correlation with massive accumulation of recombinant proteins [4]. This observation raises the possibility that Rubisco acts as a protein buffer to maintain plant homeostasis in the transplastomic plants. However, information is lacking to decipher whether this decrease is specific of Rubisco subunits or if massive recombinant protein accumulation affects other resident proteins.

Here, we have used proteomics to characterize transplastomic tobacco lines accumulating either a *Pseudomonas fluorescens p*-hydroxyphenyl pyruvate dioxygenase (HPPD) or an *Aequorea victoria* GFP [4]. We confirm that massive recombinant protein accumulation occurred at the expense of Rubisco. Furthermore, we document that such a change in Rubisco accumulation was accompanied by a specific reorientation of plant metabolism, notably affecting CO_2_ metabolism, presumably to adapt the CO_2_ concentration within chloroplasts to the actual Rubisco concentration. The implications of these findings both in terms of biotechnological applications and physiological significance are discussed.

## Results and Discussion

**Figure 1:**
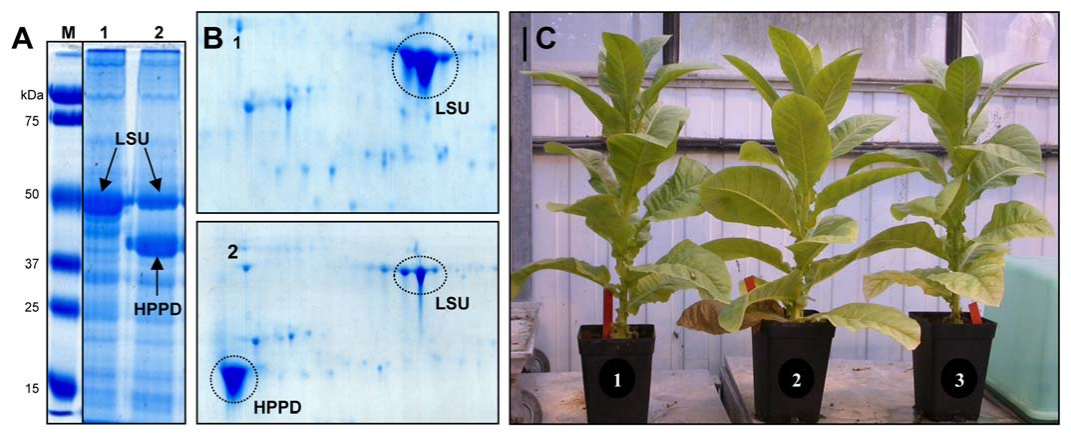
Recombinant HPPD protein accumulation and phenotype of transplastomic tobaccos. (A) Coomassie stained SDS-PAGE and (B) 2D gels of separated protein extracts from leaves of wild type plants (Lane *1* in panel *A*, Gel *1* in panel *B*) and transplastomic tobacco plants accumulating the *Pseudomonas fluorescens p*-hydroxyphenyl pyruvate dioxygenase (HPPD) (Lane *2* in panel *A*, Gel *2* in panel *B*). The position of the large subunit of Rubisco (LSU) and of the recombinant HPPD is indicated. Molecular weight markers are shown on the left of panel *A* (Lane *M*) together with their molecular weight in kDa. (C) Mature wild-type (*2*) and transplastomic plants accumulating recombinant HPPD protein (*1*,*3*) observed 15 weeks after sowing and showing an absence of phenotypic defects.

**Figure 2:**
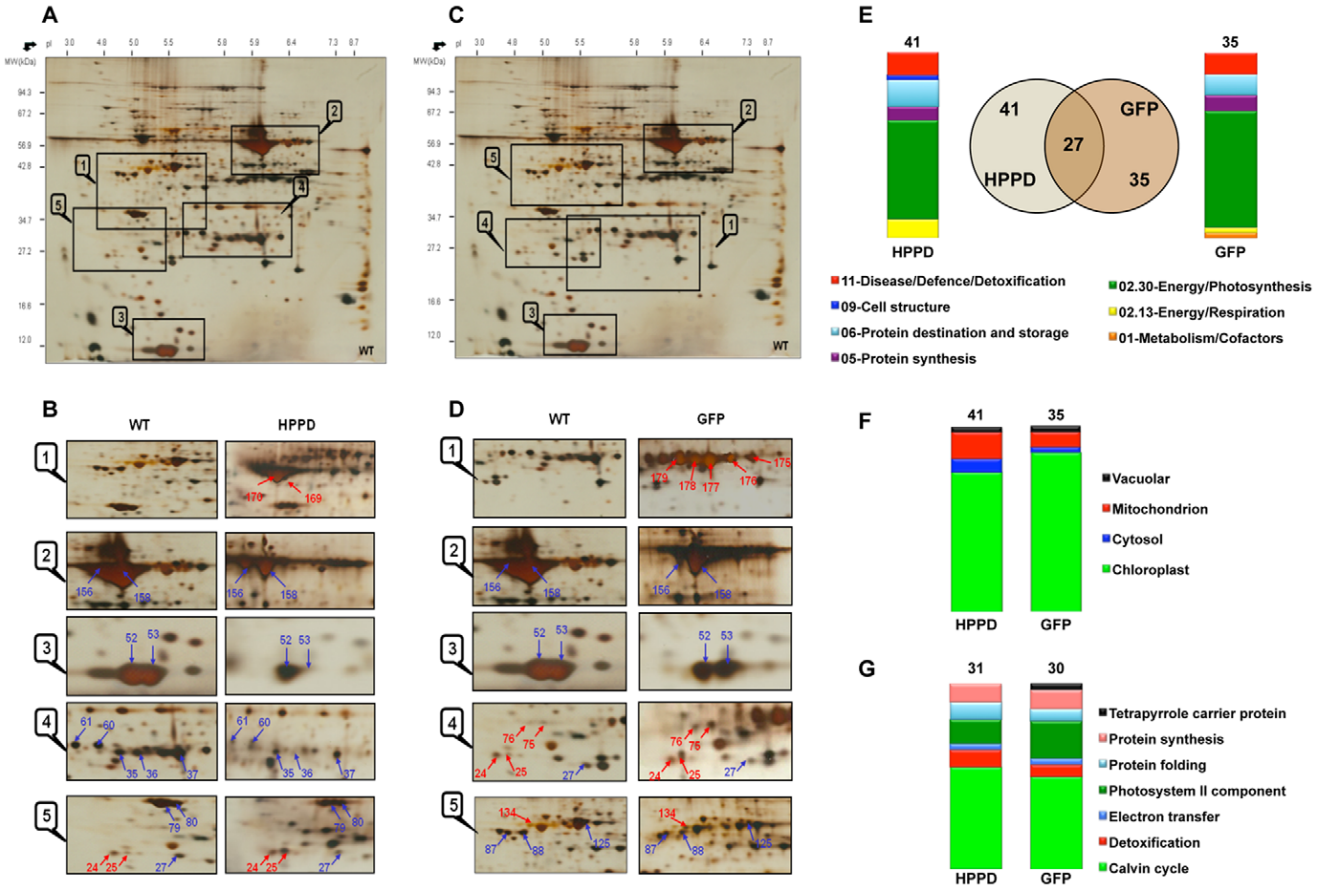
Influence of a massive recombinant protein accumulation on the proteome of tobacco leaves. (A, C) Representative silver-stained 2D gel of total soluble leaf proteins from wild type (WT) plants. (B) Enlarged windows (1–5) of 2D gels as shown in (A) for wild type plants (left) and HPPD transformants (right). (D) Enlarged windows (1–5) of 2D gels as shown in (C) for wild type plants (left) and GFP transformants (right). The numbers assigned to the proteins spots correspond to those listed in Table S2 and depicted in Figure S1. Red and blue arrows indicate protein spots that were up or down accumulated, respectively, in leaves of transplastomic tobaccos compared with wild type plants. (E) Venn diagram of varying proteins from HPPD and/or GFP transformants and functional categories assigned to the varying proteins from HPPD (41 proteins) or GFP (35 proteins) transformants, respectively. (F) Subcellular localization of the differentially expressed proteins in leaves of HPPD and GFP transformants. (G) Functional classification of varying proteins localized in chloroplasts from HPPD and GFP transformants.

The transgenic plants over accumulating the *Pseudomonas fluorescens* HPPD did not exhibit peculiar phenotypes under the growth conditions used despite the massive accumulation of the recombinant protein (Figure 1). Similar results were observed for the transgenic plants over accumulating an *Aequorea victoria* GFP (data not shown), in agreement with previous results [4]. Protein extracts were prepared from leaves of wild type and transgenic plants and were further analyzed by 2D-PAGE. The recombinant proteins were easily detected in 2D gels of leaf proteins from transgenic plants, the GFP being detected as multiple spots of similar molecular weight but differing in charge (Figures 2B and 2D). Comparative proteomics showed that out of 2170 protein spots reproducibly detected from leaf protein extracts, 54 differentially accumulated in transplastomic plants compared to wild type plants (Figure 2; Table S1 and S2; Figure S1). From LC-MS/MS analyses one spot gave two identifications, one failed to yield identification and six corresponded to the recombinant proteins (Table S1). These eight spots were excluded from quantitative analysis. Of the 46 variable spots containing unique proteins, 41 and 35 showed significant changes in plants accumulating HPPD or GFP compared to the wild type plants, respectively, of which chloroplast proteins represented the major fraction (80%) (Figure 2; Table S2).

### Recombinant protein accumulation entails numerous and specific changes in leaf proteins involved in CO_2_ metabolism

Among the 41 varying proteins identified in HPPD transformants photosynthesis proteins (54%) accounted for the major changes (Figure 2) and were mostly down accumulated (Table S2). These included the small and large Rubisco subunits (Table S2 spots 52, 53, 156 and 158; Figure 2) and several Calvin cycle enzymes such as glyceraldehyde-3-phosphate dehydrogenase (GAPDH, Table S2 spots 113 and 115), phosphoglycerate kinase (PGK, Table S2 spot 123), phosphoribulokinase (PRK, Table S2 spots 87 and 88) and carbonic anhydrase (Table S2 spots 35, 36, 37, 58, 60 and 61; Figure 2). Several of the Calvin cycle enzymes (Rubisco, PGK, PRK and GAPDH) are described as being associated in a multienzyme complex [5]. Our finding that accumulation of these enzymes is regulated in the same way in transplastomic tobacco is in excellent agreement with this proposal. Several of the Calvin cycle enzymes seem to accumulate in excess, with their *in vivo* activities modulated, as the plant physiology is not strongly affected until the content of enzymes such as Rubisco, PGK or PRK reduces to 50% or less of wild type levels [6]–[8]. Thereby, the present findings showing that decreased levels of such proteins did not result in abnormal plant phenotypes are in accordance with these previous studies.

In contrast to the decreased level in Rubisco subunits, Rubisco activase protein level was up regulated in HPPD transformants (Table S2). This enzyme modulates the activity of Rubisco by facilitating the recycling of abortive Rubisco complexes [9]. The increased level of Rubisco activase may thus contribute to the proper functioning of the Calvin cycle activity by maintaining an optimal active state of the Rubisco, thus optimizing plant performance in response to chloroplast transformation. Also, Rubisco activase has been shown to play an alternative role of chaperone, for example in helping the ribosomal complexes associated to the thylakoid membrane, in protection of the thylakoid-associated translation machinery against heat-inactivation [10], or in maintaining photosystem II (PSII) function [11]. So it is possible that Rubisco activase interacts with proteins other than Rubisco when plants are subject to modifications such as the over accumulation of alien proteins. Consistent with this, it is established that Rubisco activase is expressed in the non-photosynthetic seeds of monocots and dicots that lack Rubisco [12].

Along with such variations, this proteomic analysis revealed a down-accumulation of three protein spots corresponding to subunits of glycine decarboxylase (GDC) (Table 1; Tables S1 and S2), for which role in providing CO_2_ necessary for Rubisco functioning has been well documented [13]. GDC is involved in the photorespiratory pathway whose prime function is to reduce the toxic accumulation of phosphoglycolate produced in the light by the oxygenase activity of Rubisco. Thereby, photorespiration inhibits photosynthesis by interfering with CO_2_ fixation catalyzed by Rubisco [13]–[14]. Accordingly, a decrease in mitochondrial GDC could result in a reduction of photorespiratory flow to allow a maximum of photosynthesis activity. In agreement with this, photorespiratory bypass is accompanied by increased photosynthesis and biomass production in *Arabidopsis thaliana* [14], which could at least in part explain the fact that massive recombinant protein synthesis was not accompanied by growth defects in our present experiments. However, GDC together with serine hydroxymethyl transferase is important for the production of N^5^,N^10^-methylene-tetrahydrofolate (5,10-CH_2_-THF), a precursor of 5-CH_2_-THF involved in Met biosynthesis and the C1 metabolism [15]. Given the importance of this amino acid in the functioning of the methyl cycle, a reduction of GDC activity would be detrimental for the transformed plants. In a previous study, the amino acid composition of leaves from wild type and transformed tobaccos was assessed on the same lines as those presently used [4]. Since this analysis disclosed very similar levels of glycine and serine (the substrate and product of GDC, respectively) in wild type and transformed plants, the enzyme activity of GDC is likely to be the same in wild type and transgenic plants, suggesting that the enzyme is not affected by the massive accumulation of the recombinant protein. Furthermore, it is well known that the GDC complex is present in tremendous amounts in leaf mitochondria of higher plants [13] and there is evidence that Rubisco and GDC mRNAs cannot be translated at the same time during leaf development, presumably due to translational limitations [13]. We can thus hypothesize that GDC as Rubisco could also serve as a temporary store allowing plant cells to maintain protein homeostasis under the conditions prevailing during massive recombinant protein production. Moreover, a concomitant increase in the accumulation of serine hydroxymethyl transferase (Table 1; Tables S1 and S2) can be viewed as a mechanism to supplement GDC and to avoid altering the C1 metabolism.

**Table 1:** Soluble varying proteins from tobacco leaf protein extracts identified by LC-MS/MS involved in CO_2_ metabolism.

Spot N°	Accession number	Protein name	Organism	Subcellular localization	Function	HPPD / WT	GFP / WT
36	gi|22550386	Carbonic anhydrase	*Nicotiana tabacum*	Chloroplast	Calvin cycle	D	C
37	gi|22550386	Carbonic anhydrase	*Nicotiana tabacum*	Chloroplast	Calvin cycle	D	D
58	gi|22550386	Carbonic anhydrase	*Nicotiana tabacum*	Chloroplast	Calvin cycle	D	U
60	gi|115473	Carbonic anhydrase	*Nicotiana tabacum*	Chloroplast	Calvin cycle	D	D
61	gi|22550386	Carbonic anhydrase	*Nicotiana tabacum*	Chloroplast	Calvin cycle	D	D
35	gi|22550386	Carbonic anhydrase	*Nicotiana tabacum*	Chloroplast	Calvin cycle	D	U
92	gi|4827253	Fructose-bisphosphate aldolase	*Nicotiana paniculata*	Chloroplast	Calvin cycle	U	C
104	gi|120661	Glyceraldehyde-3-phosphate dehydrogenase	*Nicotiana tabacum*	Chloroplast	Calvin cycle	C	U
113	gi|120665	Glyceraldehyde-3-phosphate dehydrogenase	*Nicotiana tabacum*	Chloroplast	Calvin cycle	D	D
115	gi|120665	Glyceraldehyde-3-phosphate dehydrogenase	*Nicotiana tabacum*	Chloroplast	Calvin cycle	D	C
123	gi|2499497	Phosphoglycerate kinase	*Nicotiana tabacum*	Chloroplast	Calvin cycle	D	D
87	gi|115448091	Phosphoribulokinase	*Oryza sativa*	Chloroplast	Calvin cycle	D	D
88	gi|125578	Phosphoribulokinase	*Mesembryanthemum crystallinum*	Chloroplast	Calvin cycle	D	D
156	gi|4262869	Rubisco (large subunit)	*Nicotiana tabacum*	Chloroplast	Calvin cycle	D	D
158	gi|132000	Rubisco (large subunit)	*Nicotiana acuminata*	Chloroplast	Calvin cycle	D	D
52	gi|30013663	Rubisco (small subunit)	*Nicotiana tabacum*	Chloroplast	Calvin cycle	D	D
53	gi|30013663	Rubisco (small subunit)	*Nicotiana tabacum*	Chloroplast	Calvin cycle	D	D
134	gi|10720247	Rubisco activase	*Solanum pennellii*	Chloroplast	Calvin cycle	U	U
68	gi|15225249	Glycine decarboxylase (P-protein)	*Arabidopsis thaliana*	Mitochondrion	Photorespiration	D	C
109	gi|1707878	Glycine decarboxylase (T-protein)	*Solanum tuberosum*	Mitochondrion	Photorespiration	D	C
110	gi|1707878	Glycine decarboxylase (T-protein)	*Solanum tuberosum*	Mitochondrion	Photorespiration	D	D
161	gi|462187	Serine hydroxymethyltransferase	*Pisum sativum*	Mitochondrion	Photorespiration	U	C

Soluble proteins were prepared from tobacco leaves of wild type plants, and plants over accumulating a *Pseudomonas fluorescens p*-hydroxyphenyl pyruvate dioxygenase (HPPD) or *an Aequorea victoria* GFP (GFP). Proteins have been analyzed by two-dimensional electrophoresis and identified by LC-MS/MS as described under Materials and Methods. Only spots containing a single protein have been considered. For more details about the listed proteins (sequences, accumulation levels, see Tables S1 and S2). *Spot No.*, spot label; *Accession number*, accession number in NCBI database; *Protein name*, identified protein names; *Organism*, organism in which the protein has been identified; *Subcellular localization*, cellular compartment in which the protein has been identified; *Function*, protein function defined from literature; *HPPD/WT* and *GFP/WT*, patterns of variation when comparing spot volumes measured in HPPD or GFP proteome compared to wild type (WT) proteome, respectively: C, constant; D, down-accumulated; U, up-accumulated.

### Recombinant protein accumulation induces a stress response in transformed plants

The present study showed an increased accumulation of several protein spots belonging to the categories “protein destination and storage” and “disease/defense/detoxification” as various chaperones and peroxidases (Tables S1 and S2). The role of such proteins is to protect cells from various stresses [16]. In agreement with our data, these proteins have also been shown to accumulate in response to the production of recombinant proteins in bacteria [17]. Therefore it could be concluded that transgenic tobaccos over accumulating HPPD experienced a stress, as previously observed in transgenic plants exhibiting a decrease in Rubisco [16], [18]. This finding must be considered to maintain plant fitness in future studies aiming at producing recombinant proteins of interest through chloroplast transformation, and also with regards to food safety and nutritional equivalence of genetically engineered crop plants [19].

### Proteomics of GFP transformants

Since HPPD is naturally present in plants, being involved in the synthesis of plastoquinones [20], the observed proteome changes could possibly be induced by specific modifications of the plant metabolism entailed by the over accumulation of recombinant HPPD. That this was not the case is indicated by the fact that essentially similar results as those obtained for HPPD transformants were obtained with transplastomic plants accumulating the *Aequorea victoria* GFP (Figure 2; Table 1; Tables S1 and S2). Thus we conclude that the specific changes observed in the proteome of transplastomic plants do not depend on the nature of the expressed recombinant proteins.

### Hyper-accumulation of recombinant proteins in leaves does not affect the seed proteome

It is known that Rubisco has a dual role in plants, first in carbon fixation, and second as a dynamic nitrogen store during leaf senescence, when leaf proteins are remobilized for seed filling [21]–[23]. In addition to a strong down accumulation of Rubisco our study disclosed proteome variations of the transgenic plants similar to those characterizing nitrogen remobilization during leaf senescence, such as an up accumulation in cysteine protease but also a decrease in PSII components, and an increase in relative abundance of Rubisco activase and molecular chaperones [24]. The questions that arise then are i) what happens during leaf senescence in recombinant tobaccos with such a modified proteome? ii) Does the massive decrease in Rubisco alter seed quality and seed proteome? And iii) does the recombinant proteins efficiently substitute for Rubisco in seed filling? It could be anticipated that the changes caused by the over accumulation of a recombinant protein in chloroplast, by inducing a decrease in the major source of mobilizable nitrogen (Rubisco), could lead to impaired leaf senescence and altered seed filling for example due to a possible specificity of the protease(s) in charge of Rubisco remobilization. That this was not the case is shown by a comparison of the seed proteins profiles from wild type and transgenic tobaccos (Figure S2), which appeared to be very similar. Furthermore, several seed traits (e.g., number of seeds, dry weight and kinetics of germination) were nearly identical in recombinant and wild type plants (). Note also that the absence of detection of the recombinant proteins on the 2D gels of seed protein extracts is consistent with the use of plastid promoters that are mainly active in photosynthetic tissues and do not allow protein expression in seeds [25]–[26]. These results clearly indicate that the recombinant proteins were efficiently remobilized during seed filling, thereby concurring to the synthesis of seed storage proteins, as occurs normally with Rubisco. In addition to validating a limitation in protein synthesis this implies that the protease(s) responsible for Rubisco remobilization are able to mobilize the recombinant proteins. Taken together these observations can explain the invariance of the seed proteome between wild type and transgenic plants.

### Recombinant chloroplast proteome exhibits signaling-specific features

Plant cells possess three distinct genetic compartments: the nucleus, plastids and mitochondria that exchange information by anterograde signaling, from the nucleus to organelles, and retrograde signaling, from plastid and mitochondria to the nucleus [27]–[28]. Despite intensive work, retrograde signaling remains poorly understood. A well-documented example of coordination between nuclear and plastid genomes concerns the large and small subunits of Rubisco that are synthesized from the plastid and nuclear genomes [29], respectively. Our present results are in agreement with this characteristic feature (Figure 2; Table 1; Table S2) and hence lend further support to the specificity of the observed changes in protein accumulation levels. We observed that several plastidial proteins that are encoded by nuclear genes were down accumulated in response to massive accumulation of foreign proteins in the chloroplasts, including proteins from oxygen evolving complex, and Calvin cycle enzymes such as carbonic anhydrase, PRK, PGK, and GAPDH (Table S2). Thus plastidial signals entailed repression of the accumulation of several nuclear encoded proteins in response to foreign protein over accumulation in the chloroplast. This shows that the accumulation levels of Calvin cycle enzymes are subject to retrograde signaling, a finding supportive of the existence of a Calvin cycle multienzyme complex whose proper functioning presumably depends on structural (e.g., subunit stoichiometry) and/or functional (e.g., channeling regulation of the successive acting enzymes) requirements. In contrast, some nuclear encoded chloroplast proteins appeared to be up accumulated as for Rubisco activase, peroxidases or chaperonin 21. Interestingly, our study also unraveled a change in the accumulation of nuclear encoded mitochondrial proteins belonging to the GDC complex. The involvement of mitochondria in a retrograde signaling with the nucleus has already been described in non-photosynthetic organisms, notably for yeasts in response to stress or mutations that damaged the organellar functions [30]–[31] and for mammals under various physiological conditions such as aging, diet, temperature or exercise [32]–[33]. In plants much less is known [28]–[34] apart from studies documenting that alterations in the expression of nuclear genes encoding proteins of the mitochondrial electron transport chain induce the expression of nuclear genes encoding proteins involved in recovery of mitochondrial functions [35]. Also, a cross talk between the plastidial and mitochondrial genomes, in coordination with the nuclear genome, was hypothesized based on the characterization of mitochondrial mutants affected in chloroplast properties [36]. In support of this proposal, our study shows that a modification of the plastidial proteome (e.g., the down accumulation of Rubisco large subunit) leads to a nucleus response (e.g., leading to the down accumulation of the Rubisco small subunit and modification in the accumulation levels of many other enzymes, see above), which is reflected both in plastids and in mitochondria (e.g., the modification in the accumulation of GDC subunits). Hence, our findings are in agreement with the recent proposal that productive formation of electron transfer protein complexes present in animal and plant cell organelles is an important determinant of tonic retrograde signaling to the nucleus and anterograde responses influencing protective and cell death pathways [37].

In conclusion our work strongly suggests the existence of mechanisms limiting protein synthesis in plants. This finding is not without precedent. For example a recent work performed on the green alga *Chlamydomonas* showed that the expression of heterologous proteins in their chloroplasts is limited and points out the translation process as the main limiting step [38]. Previous work demonstrated that one way to enhance the accumulation of a recombinant protein (e.g., the *Phaseolus vulgaris* Arc5 arcelin) in seed tissues is to simultaneously reduce the accumulation of an abundant resident storage protein [39]. Also, a proteomic analysis performed on transgenic rice (*Oryza sativa*) seeds over accumulating a human therapeutic protein reported a decrease in endogenous storage proteins [40]. Furthermore, a recent proteomic analysis showed that the suppression of the synthesis of the glycinin and conglycinin major seed storage proteins in soybeans entails a rebalancing of protein content largely resulting from the selective increase of only a few proteins [41]. Hence, in agreement with our present findings, these results showed that soybeans can make large adjustments to their proteome and compensate for the alteration in the accumulation levels of abundant proteins by the selective modification in accumulation levels of other proteins that maintains normal protein content [41]. Proteomics thereby constitutes a powerful tool to study the impact of massive recombinant protein accumulation in chloroplast on plant physiology and metabolism. This approach contributes to improve our understanding on the plasticity of plant metabolism but also provides new avenues to evaluate possible bottlenecks in recombinant protein technology as limitations in amino acid biosynthesis, protein translation and stability. The present work also illustrates the usefulness of proteomics to assess the substantial equivalence [42] of genetically modified crops [43]–[44].

## Materials and Methods

### Plant material

Homoplasmic transgenic tobacco lines (PBD6 cultivar) expressing the *Pseudomonas fluorescens p*-hydroxyphenyl pyruvate dioxygenase (HPPD) or the *Aequorea victoria* GFP genes were as described [4]. Briefly, the transformation vector pCLT111 (GenBank CQ830291) expressing HPPD and the transformation vector pCLT 554 (GenBank EU870886) expressing GFP, target the integration of the transgenes to the same site, between the *rbcL* and *accD* tobacco plastid genes. The HPPD coding region was placed under the control of the strong tobacco plastid promoter *psbA*, and the GFP coding region was placed under the control of the corn *16S rDNA* plastid promoter. Plastid transformation was carried out according to the procedure described by Svab and Maliga [45]: the abaxial side of 4/5 week-old *in vitro* leaves, measuring 3–5 cm, were bombarded with DNA-coated gold particles using a helium-driven particle influx generator gun [46]. Transformed plants were regenerated at 24°C on Murashige and Skoog medium [47] supplemented with hormones, 6-benzylaminopurine (2 mg/L) and 1-naphtalene acetic acid (0.5 mg/L) for 2 days. The treated leaves were then cut into squares of on average 1 cm length, and the selection of transformants performed with 500 mg/L of spectinomycin hydrochloride. Explants were subcultured on fresh selection medium changed every 10 days. Resistant shoots obtained after 4 to 6 weeks were isolated and transferred to hormone free medium for regeneration and rooting before transfer to the greenhouse (natural light supplemented 16 h per day by sodium lamps providing 110 µE.m^−2^.s^−1^).

### Germination experiments

Germination assays were carried out on three replicates of 100 seeds and independent experiments for seeds from each recombinant tobacco. Seeds were incubated at 25°C, with 8-h light daily, on three sheets of absorbent paper (Roundfilter paper circles, Ø 45 mm, Schleicher and Schuell) and a black membrane filter with a white grid (ME 25/31, Ø 47 mm, Schleicher and Schuell) wetted with 1.3 mL of Millipore water in covered plastic boxes. A seed was regarded as germinated when the radicle protruded through the seed coat.

### Preparation of protein extracts

Total soluble proteins were extracted from mature fully developed leaves ground in liquid nitrogen using an extraction buffer composed of 10% (w/v) trichloroacetic acid (TCA), 0.07% (v/v) 2-mercaptoethanol (2ME), in cold acetone, supplemented with protease inhibitor cocktail tablets (Roche Diagnostics, Basel, Switzerland) [48]. The mixture was incubated on ice for 20 min and the centrifuged at 4°C at 13,000 *g* for 5 min. The final supernatant was recovered for analysis. Protein concentration was determined by the Bradford method [49] using a Protein Assay Reagent Kit from Bio-Rad.

Total soluble protein extracts from dry mature seeds (150 mg) ground in liquid nitrogen were extracted at 2°C in 2.2 ml of thiourea/urea lysis buffer [50] containing 7 M urea, 2 M thiourea, 4% (w/v) 3-[(3-cholamidopropryl)dimethylammonio]-1-propanesulfonate (CHAPS), 1% (v/v) Pharmalyte pH 3–10 carrier ampholytes, 18 mM Tris-HCl, 14 mM Trizma base, protease inhibitor cocktail tablets (Roche Diagnostics, Basel, Switzerland), 53 U/mL DNase I, 4.9 Kunitz U/mL RNase A, and 0.2% (v/v) Triton X-100. After 10 min at 4°C, 14 mM dithiothreitol (DTT) was added and the protein extracts were stirred for 20 min at 4°C, then centrifuged (35,000 *g*, 10 min) at 4°C. The final supernatant corresponded to the total soluble protein extract.

### Two-dimensional gel electrophoresis (2-DE), protein quantification and protein identification by mass spectrometry

Proteins samples were analyzed by 2-DE as described [51]. Protein extracts from tobacco leaves or seeds were separated using gel strips forming an immobilized non-linear gradient from pH 3 to pH 10 (Immobiline Dry Strip pH 3–10 NL, 18 cm; Amersham Pharmacia Biotech, Freiburg, Germany). Separation in the second dimension was carried out in polyacrylamide gels (10% (w/v) acrylamide, 0.33% (w/v) piperazidine diacrylamide, 0.18 M Trizma base, 0.1 M HCl, 0.07% (w/v) ammonium persulfate and 0.035% (v/v) Temed) as described [52]. Following silver-nitrate staining of the 2D gels, quantification of spots and comparative analysis were performed with the Image Master 2-D Elite software (Amersham Biosciences) as described [52]. For each condition analyzed 2D gels were made at least in triplicate and for a minimum of three independent extractions performed from three independently grown tobaccos lines. Spots whose relative accumulation level varied by at least a factor 1.5 (up or down) and p<0.05 were considered as varying spots when comparing 2D gels obtained from transformed leaves with control leaves.

Protein spots were excised from 2D gels with sterile tips and submitted to in-gel digestion with trypsin (sequencing grade; Roche Diagnostics). Extracted peptides were analyzed by tandem mass spectrometry on a nanoelectrospray ionization quadrupole time-of-flight hybrid mass spectrometer (Q-TOF Ultima; Waters Micromass) coupled with a nano-HPLC (Cap-LC; Waters). The peptide masses and sequences obtained were either matched automatically to proteins or EST in a non-redundant database (National Center for Biotechnology Information) using the Mascot MS/MS Ions Search algorithm using an error tolerant search of all significant protein hits (http://www.matrixscience.com) or blasted manually against the current databases as described [52] with the following search parameters: peptide and fragment mass tolerance ±0.1 Da, one missed cleavage maximum, minimum of 60 for the Mascot threshold score (corresponding to p<0.05) and at least two peptides per protein. Identified proteins were functionally described using the functional classification of Bevan et al. [53].

## Supporting Information

Figure S1 **Silver-stained 2D gel of total soluble leaf proteins from wild type tobacco.**
(PDF)

Figure S2 **Influence of recombinant proteins accumulation on seed proteome and germination.**
(PDF)

Table S1 List of the soluble varying proteins from tobacco leaf protein extracts identified by LC-MS/MS.
(PDF)

Table S2 Quantitative data for the normalized volumes of the unique varying spots.
(PDF)
