# Supplementary material for: Metabolic Adaptation in Transplastomic Plants Massively Accumulating Recombinant Proteins
Source: PLoS One. 2011 Sep 22;6(9):e25289. doi: 10.1371/journal.pone.0025289 (PMC3178635; doi:10.1371/journal.pone.0025289)
Supplement: Figure S1 — Silver-stained 2D gel of total soluble leaf proteins from wild type tobacco. (PDF) [file pone.0025289.s001.pdf]

Supporting Information
Bally *et al.*
Figure S1

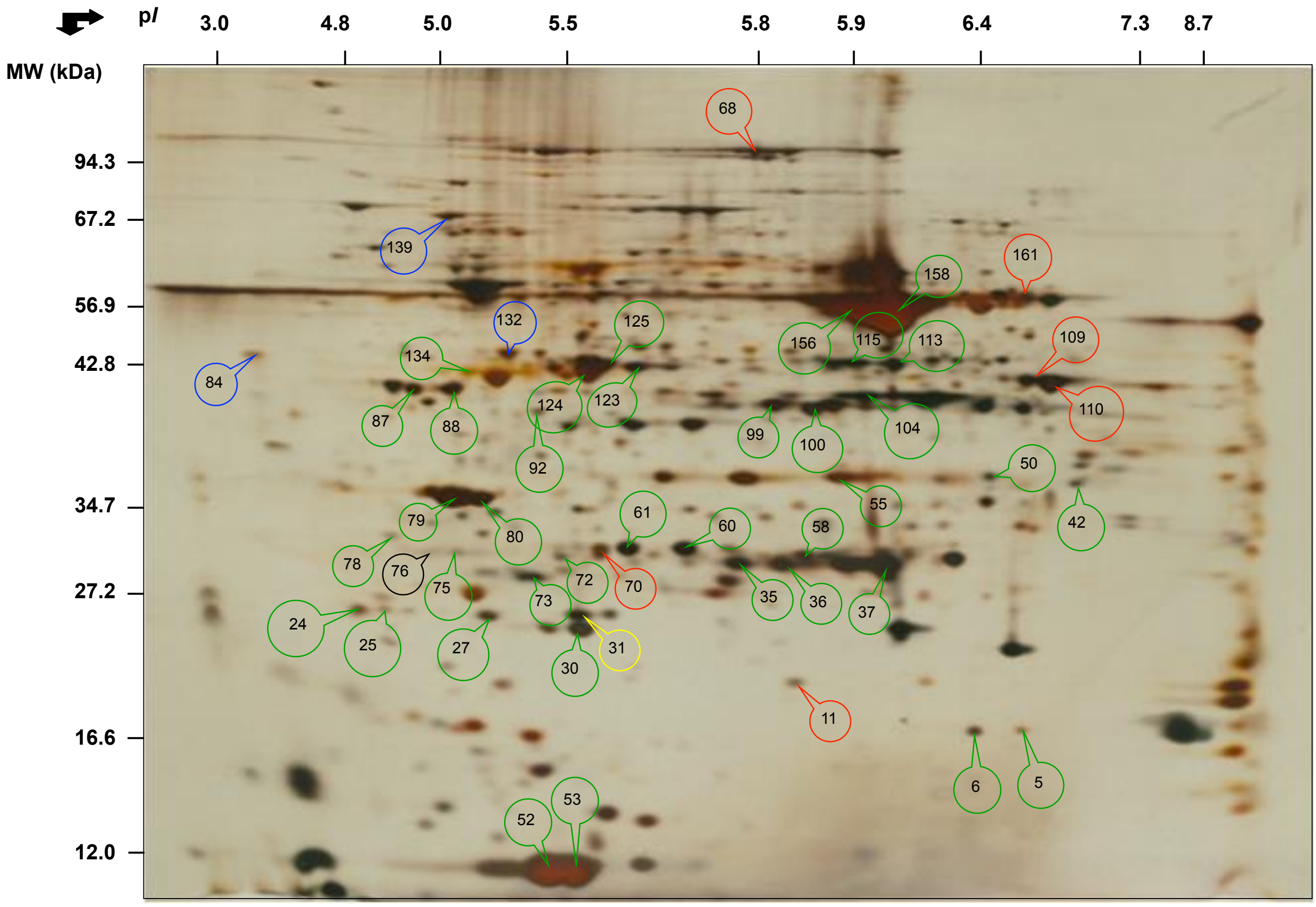

Figure S1. Silver-stained 2D gel analysis of total soluble leaf proteins (100 µg) from wild-type tobacco. Proteins identified by LC-MS/MS (**SI Table S1**) are shown. Color code: green, chloroplast proteins; blue, cytosolic proteins; black, vacuolar proteins; yellow, cell wall protein / chloroplast protein; red, mitochondrial proteins.
