# Supplementary material for: Metabolic Adaptation in Transplastomic Plants Massively Accumulating Recombinant Proteins
Source: PLoS One. 2011 Sep 22;6(9):e25289. doi: 10.1371/journal.pone.0025289 (PMC3178635; doi:10.1371/journal.pone.0025289)
Supplement: Figure S2 — Influence of recombinant proteins accumulation on seed proteome and germination. (PDF) [file pone.0025289.s002.pdf]

Supporting Information

Bally *et al.*

Figure S2

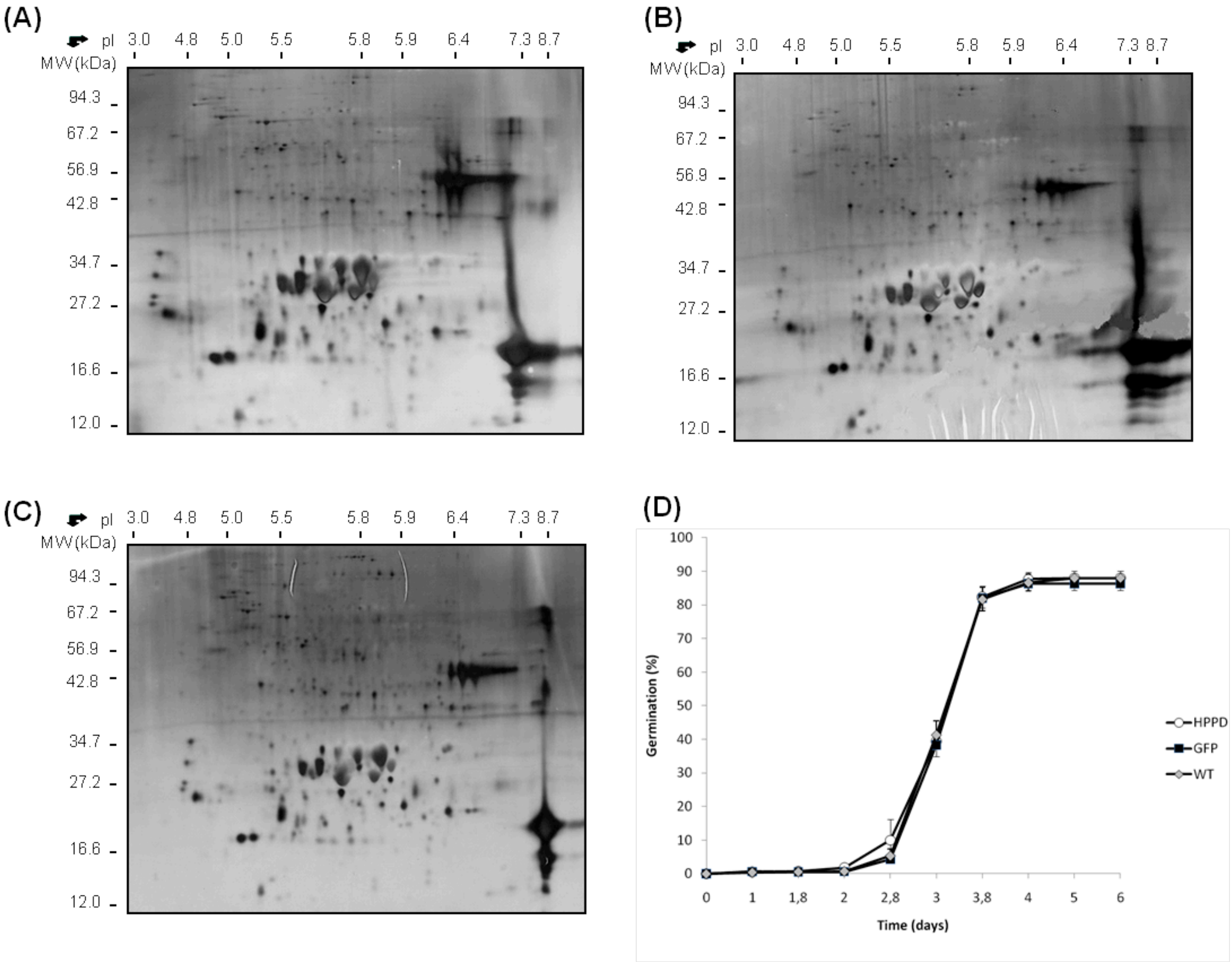

Figure S2. Influence of recombinant proteins accumulation on seed proteome and germination. Silver nitrate staining of 2D gels for mature seed proteins (100 µg) from wild-type (*A*) and transplastomic plants accumulating the *Pseudomonas fluorescens p*-hydroxyphenyl pyruvate dioxygenase (HPPD) (*B*) or the *Aequorea victoria* GFP (*C*). (*D*) Germination curves from wild-type and transplastomic plants. The graph shows a representative experiment carried out three times in triplicate.
