# Supplementary material for: Metabolic Adaptation in Transplastomic Plants Massively Accumulating Recombinant Proteins
Source: PLoS One. 2011 Sep 22;6(9):e25289. doi: 10.1371/journal.pone.0025289 (PMC3178635; doi:10.1371/journal.pone.0025289)
Supplement: Table S1 — List of the soluble varying proteins from tobacco leaf protein extracts identified by LC-MS/MS. (PDF) [file pone.0025289.s003.pdf]

Bally et al. Supplemental Information Table S1

Table S1: List of the soluble varying proteins from tobacco leaf protein extracts identified by LC/MS-MS.

Soluble proteins were prepared from tobacco leaves (wild-type (WT), overaccumulating a *Pseudomonas fluorescens p*-hydroxyphenyl pyruvate dioxygenase (HPPD) or an *Aequorea victoria* GFP (GFP)). Proteins have been analyzed by two-dimensional electrophoresis and identified by LC/MS-MS as described under Materials and Methods.

Spot No., spot label;
Number of id per spot, number of proteins identified in the corresponding spot;
Accession number, accession number in NCBI database;
Protein name, identified protein names;
Organism,organism in which the protein has been identified;
Subcellular localization, cellular compartment in which the protein has been identified;
Function, protein function defined from litterature;
Functional cattegories, functional categories defined according to the ontological classification of Bevan et al. (Bevan et al. (1998) Analysis of 1.9 Mb of contiguous sequence from chromosome 4 of *Arabidopsis thaliana*. *Nature* 391: 485-488);
% cov, Mascot coverage %, percentage of sequence coverage obtained with identified peptides with Mascot software for the orthologous protein;
Score, >60 (pep> 20), Mascot Score, identification score obtained with Mascot software;
Theo MW (Da), theoretical molecular weight;
Theo pl, theoretical isoelectric point;
Real Mascot matched queries, unique peptides identified with Mascot software;
Sequences, list of peptides identified with Mascot software.

Spot N°	Number of id per spot	Accession number	Protein name	Organism	Subcellular localization	Function	Functional categories	% cov	Score	Theo. MW	Theo. Pi	Real Mascot matched queries	Sequences
5	1	gi|47507010	Peptidylprolyl isomerase	*Nicotiana benthamiana*	Chloroplast	Protein folding	06. Protein destination and storage	14	186	22302	9,59	3	K.TPWLDER.H<br>K.LIESQETDR.G<br>R.VVISDCGELPVA.- + Carbamidomethyl (C)<br>K.RVVISDCGELPVA.- + Carbamidomethyl (C)
6	1	gi|47507010	Peptidylprolyl isomerase	*Nicotiana benthamiana*	Chloroplast	Protein folding	06. Protein destination and storage	14	174	22302	9,59	3	K.TPWLDER.H<br>K.LIESQETDR.G<br>R.VVISDCGELPVA.- + Carbamidomethyl (C)<br>K.RVVISDCGELPVA.- + Carbamidomethyl (C)
11	1	gi|76866940	Peroxiredoxin (thioredoxin peroxidase)	*Nicotiana tabacum*	Mitochondrion	Detoxification	11.06-Disease /Defence / Detoxification	14	180	33542	8,89	4	R.SWDEGVSSK.F<br>K.VSGGDVILGQI.-<br>R.WSAYVDDGNVK.V<br>K.VLNVEEAPSDFK.V
20	0		Unidentified										
24	1	gi|21912927	Peroxiredoxin (thioredoxin peroxidase)	*Nicotiana tabacum*	Chloroplast	Detoxification	11.06-Disease /Defence / Detoxification	14	116	29818	8,2	3	R.SVDETLR.T<br>K.SYNVLIPDQGIALR.G<br>K.SGGLGDLNYPLISDVTK.S
25	1	gi|92011878	Peroxiredoxin (thioredoxin peroxidase)	*Nicotiana tabacum*	Chloroplast	Detoxification	11.06-Disease /Defence / Detoxification	11	140	31932	6,49	3	R.SVDETLR.T<br>R.ASSEVPLVGNK.A<br>K.SYNVLIPDQGIALR.G
27	1	gi|19898	Photosystem II oxygen-evolving complex (23-kDa polypeptide)	*Nicotiana tabacum*	Chloroplast	Photosystem II component	02.30-Energy / Photosynthesis	14	120	27866	7,03	3	K.EVEFPGQVLR.Y<br>K.FVENAATSFSVA.-<br>R.KFVENAATSFSVA.-<br>R.KFVENAATSFSVA.- + [deamidation at N5]<br>K.ENTDFLAYNGDGFK.L + [+deamidation at N9]
30	1	gi|19898	Photosystem II oxygen-evolving complex (23-kDa polypeptide)	*Nicotiana tabacum*	Chloroplast	Photosystem II component	02.30-Energy / Photosynthesis	8	125	27866	7.03	2	K.EVEFPGQVLR.Y<br>K.FVENAATSFSVA.-<br>R.KFVENAATSFSVA.-
31	2	gi|83417162	Superoxide dismutase	*Capsicum chinense*	Cell wall	Detoxification	11.06-Disease /Defence / Detoxification	9	126	28764	6.75	2	K.GPESPAGYSCK.S + Carbamidomethyl (C)<br>K.LVEATTFLDEATIK.K
	2	gi|76870069	Peptide methionine sulfoxide reductase	*Nicotiana tabacum*	Chloroplast	Protein folding	06. Protein destination and storage		96	26925	5.44	2	R.QGNDVGTQYR.S<br>R.NIVTEILPAKK.F
35	1	gi|22550386	Carbonic anhydrase	*Nicotiana tabacum*	Chloroplast	Calvin cycle	02.30-Energy / Photosynthesis	13	195	34431	6.19	4	K.NPALYGELSK.G<br>K.YMVFACSDSR.V + Carbamidomethyl (C)<br>K.YMVFACSDSR.V + Carbamidomethyl (C); Oxidation (M)<br>R.NIANMVPAYDK.T + Oxidation (M)<br>K.CFADQCTACEK.E + 3 Carbamidomethyl (C)
36	1	gi|22550386	Carbonic anhydrase	*Nicotiana tabacum*	Chloroplast	Calvin cycle	02.30-Energy / Photosynthesis	13	196	34431	6.19	4	K.NPALYGELSK.G<br>K.YMVFACSDSR.V + Carbamidomethyl (C)<br>K.YMVFACSDSR.V + Carbamidomethyl (C); Oxidation (M)<br>R.NIANMVPAYDK.T + Oxidation (M)<br>K.CFADQCTACEK.E + 3 Carbamidomethyl (C)
37	1	gi|22550386	Carbonic anhydrase	*Nicotiana tabacum*	Chloroplast	Calvin cycle	02.30-Energy / Photosynthesis	16	374	34431	6.19	5	K.NPALYGELSK.G

													K.YMVFACSDSR.V + Carbamidomethyl (C)
													K.YMVFACSDSR.V + Carbamidomethyl (C); Oxidation (M)
													R.NIANMVPAYDK.T + Oxidation (M)
													K.CFADQCTACEK.E + 3 Carbamidomethyl (C)
													R.NIANMVPAYDKTR.Y
													K.YEKNPALYGELSK.G
													K.NPALYGELSKGQSPK.Y
42	1	gi|5381255	Peroxidase	*Nicotiana tabacum*	Chloroplast	Detoxification	11.06-Disease /Defence / Detoxification	5	76	35709	7.59	2	K.GFDVIEDAK.T
													R.AESIVQSTVR.A
50	1	gi|11465970	Cytochrome f	*Nicotiana tabacum*	Chloroplast	Electron transfer (cytochrome *b6f* complex)	02.30-Energy / Photosynthesis	18	247	35224	9.12	5	R.GQIYPDGSK.S
													R.IPYDMQLK.Q + Oxidation (M)
													K.NILVIGPVPGQK.Y
													K.GGYEITITDASDGR.Q
													K.SNNTVYNATAAGIVSK.I
52	1	gi|30013663	Ribulose-1,5-bisphosphate carboxylase (small subunit)	*Nicotiana tabacum*	Chloroplast	Calvin cycle	02.30-Energy / Photosynthesis	34	425	20268	7.57	6	K.SPGYYDGR.Y
													K.AYPEAWIR.I
													R.GFVYRENNK.S
													K.KAYPEAWIR.I
													R.ENNKSPGYYDGR.Y
													R.ENNKSPGYYDGR.Y
													K.NGWVPCLEFETER.G + Carbamidomethyl (C)
													R.QVQCISFIAYKPEGY.- + Carbamidomethyl (C); [Pyroglutamic Acid at N-term Q]
													R.QVQCISFIAYKPEGY.- + Carbamidomethyl (C)
													R.QVQCISFIAYKPEGY.- + Carbamidomethyl (C)
53	1	gi|30013663	Ribulose-1,5-bisphosphate carboxylase (small subunit)	*Nicotiana tabacum*	Chloroplast	Calvin cycle	02.30-Energy / Photosynthesis	13	153	20268	7.57	4	K.SPGYYDGR.Y
													K.AYPEAWIR.I
													R.GFVYRENNK.S
													R.ENNKSPGYYDGR.Y
													R.ENNKSPGYYDGR.Y
58	1	gi|22550386	Carbonic anhydrase	*Nicotiana tabacum*	Chloroplast	Calvin cycle	02.30-Energy / Photosynthesis	13	241	34431	6.19	4	K.NPALYGELSK.G
													K.YMVFACSDSR.V + Carbamidomethyl (C)
													K.YMVFACSDSR.V + Carbamidomethyl (C); Oxidation (M)
													R.NIANMVPAYDK.T + Oxidation (M)
													K.CFADQCTACEK.E + 3 Carbamidomethyl (C)
													R.NIANMVPAYDKTR.Y
60	1	gi|115473	Carbonic anhydrase	*Nicotiana tabacum*	Chloroplast	Calvin cycle	02.30-Energy / Photosynthesis	29	413	34489	6.41	8	K.IGLPAKAK.V
													K.GELGPIAAAR.V
													K.NPALYGELSK.G
													K.FMVFACSDSR.V + Carbamidomethyl (C)
													K.FMVFACSDSR.V + Carbamidomethyl (C); Oxidation (M)
													R.NIANMVPAYDK.T + Oxidation (M)
													K.ESYEQAIAALEK.L
													K.CFADQCTACEK.E + 3 Carbamidomethyl (C)
													R.NIANMVPAYDKTR.Y
													R.NIANMVPAYDKTR.Y
													R.NIANMVPAYDKTR.Y
													R.NIANMVPAYDKTR.Y + Oxidation (M)
													R.NIANMVPAYDKTR.Y + Oxidation (M)
													R.VDQITAELQSSDGSK.P
													K.NPALYGELSKGQSPK.F
61	1	gi|22550386	Carbonic anhydrase	*Nicotiana tabacum*	Chloroplast	Calvin cycle	02.30-Energy / Photosynthesis	19	403	34431	6.19	6	K.GELGPIAAAR.V
													K.NPALYGELSK.G
													K.YMVFACSDSR.V + Carbamidomethyl (C)
													K.YMVFACSDSR.V + Carbamidomethyl (C); Oxidation (M)

													R.NIANMVPAYDK.T + Oxidation (M)<br>K.CFADQCTACEK.E + 3 Carbamidomethyl (C)<br>R.NIANMVPAYDKTR.Y<br>R.NIANMVPAYDKTR.Y<br>K.LLSEKGELGPIAAAR.V<br>K.NPALYGELSKGQSPK.Y
68	1	gi|15225249	Glycine decarboxylase (P-protein)	*Arabidopsis thaliana*	Mitochondrion	Photorespiration	02.13-Energy / Respiration	4	294	113703	6.18	5	K.IVAVGTDAK.G<br>R.VDNVYGDR.N<br>K.GNINIEELR.N<br>R.IIGVSVDSSGK.Q<br>K.IAILNANYMAK.R<br>K.IAILNANYMAK.R + Oxidation (M)
70	1	gi|559005	Ascorbate peroxidase	*Nicotiana tabacum*	Mitochondrion	Detoxification	11.06-Disease /Defence / Detoxification	19	272	27371	5.43	5	K.TEPPVEGR.L<br>K.TGGPFGTMR.L<br>K.TGGPFGTMR.L + Oxidation (M)<br>K.NCAPLMLR.L + Carbamidomethyl (C)<br>K.NCAPLMLR.L + Carbamidomethyl (C); Oxidation (M)<br>K.EGLLQLPSDK.A<br>M.GKCYPTVSEEYLK.A + Carbamidomethyl (C)
72	1	gi|92037383	SOUL heme-binding family protein	*Arabidopsis thaliana*	Chloroplast	Tetrapyrrole carrier protein	01.07-Metabolism / Cofactors	11	157	32673	7.74	3	R.LFDYIQGK.N<br>K.EAAALSASIADTK.W<br>K.EAAALSASIADTK.W<br>R.IECPNYDLIQSGK.D + Carbamidomethyl (C)
73	1	gi|7331143	Chaperonin 21	*Lycopersicon esculentum*	Chloroplast	Protein folding	06. Protein destination and storage	12	192	26546	6.85	3	K.DLQPLNDR.V<br>K.TAGGLLLTEAAK.E<br>K.YAGTEVEFDGSK.H
75	1	gi|11465949	Photosystem II protein D2	*Nicotiana tabacum*	Chloroplast	Photosystem II component	02.30-Energy / Photosynthesis	6	62	39510	5.33	2	R.AYDFVSQEIR.A<br>R.AAEDPEFETFYTK.N
76	1	gi|8347420	Cysteine protease	*Nicotiana tabacum*	Vacuolar	Proteolysis	06. Protein destination and storage	6	68	39175	6.95	2	K.FSSENVGVK.V<br>K.SNGGLDTEEAYPYTGK.N
78	1	gi|11134054	Photosystem II oxygen-evolving complex (33-kDa polypeptide)	*Nicotiana tabacum*	Chloroplast	Photosystem II component	02.30-Energy / Photosynthesis	14	340	35206	5.89	5	R.GSSFLDPK.G<br>R.GSSFLDPKGR.G<br>R.LTFDEIQSK.T<br>K.NAPPDFQKTK.L<br>K.RLTFDEIQSK.T<br>R.GGSTGYDNAVALPAGGR.G<br>R.GGSTGYDNAVALPAGGR.G + [+deamidation at N8]
79	1	gi|147791852	Photosystem II oxygen-evolving complex (33-kDa polypeptide)	*Vitis vinifera*	Chloroplast	Photosystem II component	02.30-Energy / Photosynthesis	17	352	33213	5.87	6	R.GSSFLDPK.G<br>R.GDEEELKK.E<br>R.GSSFLDPKGR.G<br>R.LTYDEIQSK.T<br>K.RLTYDEIQSK.T<br>K.RLTYDEIQSK.T<br>K.FCLEPTSFTVK.A + Carbamidomethyl (C)<br>R.GGSTGYDNAVALPAGGR.G
80	1	gi|11134054	Photosystem II oxygen-evolving complex (33-kDa polypeptide)	*Nicotiana tabacum*	Chloroplast	Photosystem II component	02.30-Energy / Photosynthesis	21	477	35367	5.84	9	R.GSSFLDPK.G<br>K.NAPPDFQK.T<br>R.GDEEELQK.E<br>R.GSSFLDPKGR.G<br>R.LTFDEIQSK.T<br>K.NAPPDFQKTK.L<br>K.RLTFDEIQSK.T<br>K.FCLEPTSFTVK.A + Carbamidomethyl (C)<br>K.AESVNKNAPPDFQK.T

													R.GGSTGYDNAVALPAGGR.G
84	1	gi|13310811	Ankyrin-repeat protein HBP1	*Nicotiana tabacum*	Cytosol	Plasmodesmal gating	06. Protein destination and storage	16	232	37294	4.45	5	K.LNNQQEVLK.L
													K.CAQILLEAGAK.V + Carbamidomethyl (C)
													R.YWNDQETLK.K
													K.SSESSSTEAPSGEAR.T
													K.SSESSSTEAPSGEAR.T
													M.SEGEKVLPTASADEK.S + [+acetylation at N-term S]
87	1	gi|115448091	Phosphoribulokinase	*Oryza sativ*	Chloroplast	Calvin cycle	02.30-Energy / Photosynthesis	13	323	44837	5.68	6	K.GVTALDPR.A
													R.GHSLESIK.A
													K.EKGVTALDPR.A
													R.LTSVFGGAAEPPK.G
													R.LTSVFGGAAEPPK.G
													K.FYGEVTQQMLK.H
													K.FYGEVTQQMLK.H + Oxidation (M)
													R.ANDFDLMYEQVK.A
													R.ANDFDLMYEQVK.A + Oxidation (M)
88	1	gi|125578	Phosphoribulokinase	*Mesembryanthemum crystallinum*	Chloroplast	Calvin cycle	02.30-Energy / Photosynthesis	18	434	44086	6.03	8	K.GVTALDPR.A
													R.GHSLESIK.A
													K.LTCSYPGIK.F + Carbamidomethyl (C)
													K.EKGVTALDPR.A
													R.LTSVFGGAAEPPR.G
													K.FYGEVTQQMLK.H
													K.FYGEVTQQMLK.H + Oxidation (M)
													R.KPDFDAYIDPQK.Q
													R.ANDFDLMYEQVK.A
													R.ANDFDLMYEQVK.A + Oxidation (M)
92	1	gi|4827253	Fructose-bisphosphate aldolase	*Nicotiana paniculata*	Chloroplast	Calvin cycle	02.30-Energy / Photosynthesis	14	219	42789	6.38	6	K.EAAWGLAR.Y
													K.ANSLAQLGK.Y
													R.ALQNTCLK.T + Carbamidomethyl (C)
													K.AAQDALLTR.A
													R.TAAYYQQGAR.F
													R.LASIGLENTEANR.Q
99	1	gi|32746733	Chloroplast mRNA-binding protein	*Nicotiana tabacum*	Chloroplast	Protein synthesis	05.04-Protein synthesis / Translation factors	10	206	44217	6.51	4	K.ADAGHVGVEK.Y
													R.FSEITGAGGR.T
													K.ILEALKVPVAA.-
													R.TIWGDPADVGK.I
100	1	gi|147773479	Chloroplast mRNA-binding protein	*Vitis vinifera*	Chloroplast	Protein synthesis	05.04-Protein synthesis / Translation factors	13	230	42304	8.74	5	K.QVFNISGEK.Y
													K.QVFNISGEK.Y
													K.QVFNISGEK.Y
													K.LETESLLDSR.G + [+methylation at S9]
													K.EADFSTDDIILGK.S
													R.KEADFSTDDIILGK.S
													K.GAGFPEPEIVHYNPK.E
104	1	gi|120661	Glyceraldehyde-3-phosphate dehydrogenase	*Nicotiana tabacum*	Chloroplast	Calvin cycle	02.30-Energy / Photosynthesis	20	364	41837	6.6	8	K.VLITAPGK.G
													R.LLDASHR.D
													K.VAINGFGR.I
													R.EAADKELK.G
													K.VIQVVSDR.N
													K.TFAEEVNAAFR.E
													R.AAALNIVPTSTGAAK.A
													K.DSPLDVIAINDTGGVK.Q
109	1	gi|1707878	Glycine decarboxylase (T-protein)	*Solanum tuberosum*	Mitochondrion	Photorespiration	02.13-Energy / Respiration	20	356	44249	8.77	4	R.KEQCLALGTR.L + Carbamidomethyl (C)
													K.DGVYPEKVNAGR.Q
													K.DGVYPEKVNAGR.Q

													K.EQIFEMPTGGAAIMR.E + 2 Oxidation (M)
													K.VTNDHIYLVVNAGCR.D + Carbamidomethyl (C)
110	1	gi|1707878	Glycine decarboxylase (T-protein)	*Solanum tuberosum*	Mitochondrion	Photorespiration	02.13-Energy / Respiration	22	497	44249	8.77	9	K.QIEEGPK.I
													K.MPFVPTK.Y + Oxidation (M)
													K.NIAMGYVK.T + Oxidation (M)
													K.NIAMGYVK.T + Oxidation (M)
													K.MYFGEFR.V
													K.MYFGEFR.V + Oxidation (M)
													K.ALLEKSEGK.I
													R.VGFFSSGPPPR.S
													R.RVGFFSSGPPPR.S
													R.RVGFFSSGPPPR.S
													R.RVGFFSSGPPPR.S
													R.AEGGFLGAEVILK.Q
													K.TVLYDFHVVNGGK.M
													R.RAEGGFLGAEVILK.Q
													K.VTNDHIYLVVNAGCR.D + Carbamidomethyl (C)
113	1	gi|120665	Glyceraldehyde-3-phosphate dehydrogenase	*Nicotiana tabacum*	Chloroplast	Calvin cycle	02.30-Energy / Photosynthesis	20	542	47440	8.83	8	R.LLDASHR.D
													K.VIITAPAK.G
													K.KVIITAPAK.G
													K.YDSMLGTFK.A
													K.AVSLVLPQLK.G
													K.GITAEDVNAAFR.K
													K.IVDNETISVDGK.H
													R.AAALNIVPTSTGAAK.A
													K.DSPLDVVVVNDSGGVK.N
115	1	gi|120665	Glyceraldehyde-3-phosphate dehydrogenase	*Nicotiana tabacum*	Chloroplast	Calvin cycle	02.30-Energy / Photosynthesis	28	755	47440	8.83	14	R.LLDASHR.D
													K.VIITAPAK.G
													K.KVIITAPAK.G
													K.AVSLVLPQLK.G
													K.VMDEELGIVK.G
													K.VMDEELGIVK.G + Oxidation (M)
													K.AVSLVLPQLKGK.L
													K.GITAEDVNAAFR.K
													K.IVDNETISVDGK.H
													R.AAALNIVPTSTGAAK.A
													K.YDSMLGTFKADVK.I
													K.YDSMLGTFKADVK.I
													K.GTMTTTHSYTGDQR.L + Oxidation (M)
													K.DSPLDVVVVNDSGGVK.N
													R.ARAAALNIVPTSTGAAK.A
													K.IVDNETISVDGKHIK.V
													R.KDSPLDVVVVNDSGGVK.N
123	1	gi|2499497	Phosphoglycerate kinase	*Nicotiana tabacum*	Chloroplast	Calvin cycle	02.30-Energy / Photosynthesis	30	808	50146	8.48	14	K.KLADLSGK.G
													K.FAPDANSK.I
													K.YSLAPLVPR.L
													K.RPFAAIVGGSK.V
													K.SVGDLTAAELK.G
													K.TFNDALDTTK.T
													R.LSELLGIQVVK.V
													K.VILSSHLGRPK.G
													K.SVGDLTAAELKGK.K
													K.SVGDLTAAELKGK.K
													K.ELDYLVGAVSNPK.R

													K.GVTTIIGGGDSVAAVEK.V
													K.VLPGVIALDEADAPVAV.-
													R.ADLNVPLDDNQNITDDTR.I
													K.LADLSGKGVTTIIGGGDSVAAVEK.V
124	1	gi|218312	Translation elongation factor TuB	*Nicotiana sylvestris*	Chloroplast	Protein synthesis	05.04-Protein synthesis / Translation factors	18	366	46672	5.7	6	K.VGEIVDIVGLK.D
													K.TVGAGVIQKILE.-
													R.NTTVTGVEMFQK.I
													K.KYDEIDAAPEER.A
													K.ILDEAMAGDNVGLLLR.G
													R.GITINTATVEYETENR.H
125	1	gi|68566313	Translation elongation factor TuA	*Nicotiana tabacum*	Chloroplast	Protein synthesis	05.04-Protein synthesis / Translation factors	20	689	51924	6.34	11	K.EHILLAK.Q
													K.TVGAGVIQK.I
													R.FAIREGGK.T
													R.IGDTVDIVGLK.D
													K.TVGAGVIQKIIE.-
													R.EGGKTVGAGVIQK.I
													R.STTVTGVEMFQK.I
													R.STTVTGVEMFQK.I + Oxidation (M)
													K.KYDEIDAAPEER.A
													R.IGDTVDIVGLKDTR.S
													R.IGDTVDIVGLKDTR.S
													K.ILDEAMAGDNVGLLLR.G
													K.ILDEAMAGDNVGLLLR.G + Oxidation (M)
													R.GITINTATVEYETENR.H
132	1	gi|6683504	Actin	*Mimosa pudica*	Cytosol	Cytoskeleton	09.04-Cell structure / Cytoskeleton	41	722	41703	5.31	13	K.RGILTLK.Y
													K.AGFAGDDAPR.A
													K.IKVVAPPER.K
													R.HTGVMVGMGQK.D
													K.EITALAPSSMK.I
													K.EITALAPSSMK.I + Oxidation (M)
													R.GYMFTTSAER.E + Oxidation (M)
													K.DAYVGDEAQSK.R
													R.AVFPSIVGRPR.H
													K.DAYVGDEAQSKR.G
													K.EITALAPSSMKIK.V
													K.GEYDESGPSIVHR.K
													R.LDLAGRDLTDSLMK.I
													K.NYELPDGQVITIGAER.F
													K.LAYVALDYEQELETAK.T
													R.VAPEEHPVLLTEAPLNPK.A
													R.VAPEEHPVLLTEAPLNPK.A
134	1	gi|10720247	RuBisCO activase	*Solanum pennellii*	Chloroplast	Calvin cycle	02.30-Energy / Photosynthesis	18	500	50669	8.61	12	R.EAAEIIR.K
													R.VYDDEVR.K
													F.INDLDAGAGR.M
													R.VYDDEVRK.W
													R.YREAAEIIR.K
													R.ARVYDDEVR.K
													K.VPLILGVWGGK.G
													R.TDNVPEEAVVK.I
													K.RVQLAETYLK.E
													C.CLFINDLDAGAGR.M + Carbamidomethyl (C)
													K.GNMCCLFINDLDAGAGR.M + 2 Carbamidomethyl (C)
													K.LLEYGNMLVQEQENVK.R
													K.LLEYGNMLVQEQENVK.R

139	1	gi|30025966	Heat shock protein (HSP 70)	*Nicotiana tabacum*	Cytosol	Protein folding	06. Protein destination and storage	23	692	70832	5.17	14	R.NTTIPTKK.E
													R.LSKEEIEK.M
													K.ITITNDKGR.L
													R.KCMEPVEK.C + Carbamidomethyl (C)
													R.FSDASVQSDIK.L
													R.VEIIANDQGNR.T
													K.DAGVISGLNVMR.I
													K.DAGVISGLNVMR.I + Oxidation (M)
													K.NALENYAYNMR.N
													K.ELESICNPIIAK.M + Carbamidomethyl (C)
													R.TTPSYVGFTDSER.L
													R.QATKDAGVISGLNVMR.I + [Pyroglutamic Acid at N-term Q]
													R.QATKDAGVISGLNVMR.I + Oxidation (M); [Pyroglutamic Acid at N-term Q]
													K.NQVAMNPINTVFDAK.R
													K.ATAGDTHLGGEDFDNR.M
													K.NAVVTVPAYFNDSQR.Q
156	1	gi|4262869	Ribulose-1,5-bisphosphate carboxylase (large subunit)	*Nicotiana tabacum*	Chloroplast	Calvin cycle	02.30-Energy / Photosynthesis	32	840	52895	6.29	17	R.VALEACVK.A + Carbamidomethyl (C)
													R.AVYECLR.G + Carbamidomethyl (C)
													R.AMHAVIDR.Q
													K.AQAETGEIK.G
													R.ALRLEDLR.I
													K.DTDILAAFR.V
													R.DDFVEQDR.S
													K.LGLSAKNYGR.A
													R.VALEACVKAR.N + Carbamidomethyl (C)
													R.DLAQEGNEIIR.E
													K.LTYYTPEYQTK.D
													K.DDENVNSQPFMR.W
													K.TFQGPPHGIQVER.D
													K.YGRPLLGCTIKPK.L + Carbamidomethyl (C)
													R.NEGRDLAQEGNEIIR.E
													K.GHYLNATAGTCEEMIKR.A + Carbamidomethyl (C)
													R.GGLDFTKDDENVNSQPFMR.W + Oxidation (M)
158	1	gi|132000	Ribulose-1,5-bisphosphate carboxylase (large subunit)	*Nicotiana acuminata*	Chloroplast	Calvin cycle	02.30-Energy / Photosynthesis	28	668	52908	6.41	13	R.AVYECLR.G + Carbamidomethyl (C)
													R.AMHAVIDR.Q
													R.ALRLEDLR.I
													K.ASVGFKAGVK.E + [+acetyltaion at K6]
													K.DTDILAAFR.V
													R.DDFVEQDR.S
													R.DLAQEGNEIIR.E
													R.FLFCAEAIYK.A + Carbamidomethyl (C)
													K.LTYYTPEYQTK.D
													K.TFQGPPHGIQVER.D
													K.EYKLTYYTPEYQTK.D
													K.GHYLNATAGTCEEMIKR.A + Carbamidomethyl (C)
													R.GGLDFTKDDENVNSQPFMR.W
													R.GGLDFTKDDENVNSQPFMR.W
													K.LTYYTPEYQTKDTDILAAFR.V
161	1	gi|462187	Serine hydroxymethyltransferase	*Pisum sativum*	Mitochondrion	Photorespiration	02.13-Energy / Respiration	5	144	57256	8.71	4	R.LYDYAR.I
													R.GAMIFFR.K
													R.MGTPALTSR.G
													R.MGTPALTSR.G + Oxidation (M)
													K.YSEGYPGAR.Y
169	1	gi|7766898	4-Hydroxyphenylpyruvate dioxygenase	*Pseudomonas fluorescens*		Recombinant protein	Recombinant protein	40	834	40026	5.01	16	K.NVHLYR.Q

													K.TWDALKK.I
													K.GEYTGLTSK.A
													K.ALFESIER.D
													R.IPLNEESSK.G
													K.AMSAPDGMIR.I + Oxidation (M)
													K.AMSAPDGMIR.I + 2 Oxidation (M)
													L.LDGSSVEGDKR.L
													K.GIGGAPLYLIDR.F
													K.GIGGAPLYLIDR.F
													R.KGDDGFGEGNFK.A
													I.LLDGSSVEGDKR.L
													R.GILLDGSSVEGDK.R
													R.MVYWANFYEK.L
													R.MVYWANFYEK.L + Oxidation (M)
													K.VIDHLTHNVYR.G
													R.GILLDGSSVEGDKR.L
													K.ALFESIERDQVR.R
													R.QGEINLILNNEPN.S
													R.LPDHGEPVDQLQAR.G
													R.LPDHGEPVDQLQAR.G
													R.YFDIKGEYTGLTSK.A
													R.YFDIKGEYTGLTSK.A
													K.AMSAPDGMIRIPLNEESSK.G
175	1	gi|1943561	Green fluorescent protein	*Aequorea victoria*		Recombinant protein	Recombinant protein	21	316	26581	5.68	5	K.FICTTGK.L + Carbamidomethyl (C)
													K.FEGDTLVNR.I
													K.SAMPEGYVQER.T
													K.SAFPEGYVQER.T
													R.AEVKFEGDTLVNR.I
													K.FSVSGEGEGDATYGK.L
													K.FSVSGEGEGDATYGKLTLK.F
176	1	gi|150261557	Green fluorescent protein	*Aequorea victoria*		Recombinant protein	Recombinant protein	31	618	26807	5.08	8	R.YPDHMK.R + Oxidation (M)
													K.FICTTGK.L + Carbamidomethyl (C)
													R.YPDHMKR.H
													K.FEGDTLVNR.I
													K.SAMVEGYVQER.T
													K.SAMPEGYVQER.T + Oxidation (M)
													R.AEVKFEGDTLVNR.I
													K.FSVSGEGEGDATYGK.L
													K.FEGDTLVNRIELK.G
													K.GIDFKEDGNILGHK.L
													K.FSVSGEGEGDATYGKLTLK.F
													K.FSVSGEGEGDATYGKLTLK.F
													K.FSVSGEGEGDATYGKLTLK.F + [+Phosphorylation at S2]
177	1	gi|1943561	Green fluorescent protein	*Aequorea victoria*		Recombinant protein	Recombinant protein	21	418	26581	5,68	5	K.FICTTGK.L + Carbamidomethyl (C)
													K.FEGDTLVNR.I
													K.SATPEGYVQER.T
													K.SAMPEGYVQER.T + Oxidation (M)
													R.AEVKFEGDTLVNR.I
													R.AEVKFEGDTLVNR.I
													K.FSVSGEGEGDATYGK.L
													K.FSVSGEGEGDATYGKLTLK.F
													K.FSVSGEGEGDATYGKLTLK.F + [+Phosphorylation at S2]
178	1	gi|1943561	Green fluorescent protein	*Aequorea victoria*		Recombinant protein	Recombinant protein	22	451	26581	5,68	5	K.FICTTGK.L + Carbamidomethyl (C)
													K.SATPEGYVQER.T

													K.SAMPEGYVQER.T
													K.SAMPEGYVQER.T + Oxidation (M)
													R.AEVKFEGDTLVNR.I
													R.AEVKFEGDTLVNR.I
													K.FSVSGEGEGDATYGK.L
													K.FEGDTLVNRIELK.G
													K.FSVSGEGEGDATYGKLTLK.F
													K.FSVSGEGEGDATYGKLTLK.F
179	1	gi|1490529	Green fluorescent protein	*Cloning vector pGFPuv*		Recombinant protein	Recombinant protein	25	294	26779	5,8	6	K.FICTTGK.L + Carbamidomethyl (C)
													K.FEGDTLVNR.I
													K.SAMPEGYVQER.T
													K.SAMPEGYVQER.T + Oxidation (M)
													R.TISFKDDGNYK.T
													R.AEVKFEGDTLVNR.I
													K.FSVSGEGEGDATYGK.L
													K.FSVSGEGEGDATYGKLTLK.F
