# Supplementary material for: Metabolic Adaptation in Transplastomic Plants Massively Accumulating Recombinant Proteins
Source: PLoS One. 2011 Sep 22;6(9):e25289. doi: 10.1371/journal.pone.0025289 (PMC3178635; doi:10.1371/journal.pone.0025289)
Supplement: Table S2 — Quantitative data for the normalized volumes of the unique varying spots. (PDF) [file pone.0025289.s004.pdf]

Bally et al. Supplemental Information Table S2

Table S2: Quantitative data for the normalized volumes of the unique varying spots.
Soluble proteins were prepared from tobacco leaves (wild-type (WT), overaccumulating a *Pseudomonas fluorescens p*-hydroxyphenyl pyruvate dioxygenase (HPPD) or an *Aequorea victoria* GFP (GFP)).
Proteins have been analyzed by two-dimensional electrophoresis and identified by LC/MS-MS as described under Materials and Methods.
Spot No., spot label;
Number of id per spot, number of proteins identified in the corresponding spot;
Accession number, accession number in NCBI database;
Protein name, identified protein names;
Organism, organism in which the protein has been identified;
Subcellular localization, cellular compartment in which the protein has been identified;
Function, protein function defined from litterature;
Functional cattegories, functional categories defined according to the ontological classification of Bevan et al. (Bevan et al. (1998) Analysis of 1.9 Mb of contiguous sequence from chromosome 4 of Arabidopsis thaliana. Nature 391: 485-488);
Mean HPPD/Mean WT, ratio of the mean of normalized spot volumes measured in HPPD proteome compared to WT proteome;
pHPPD, p value when comparing spot volumes measured in HPPD proteome compared to WT proteome (only variations for which p<0.05 are considered);
HPPD/WT, pattern of variation when comparing spot volumes measured in HPPD proteome compared to WT proteome: C, constant; D, down-accumulated; U, up-accumulated;
Mean GFP/Mean WT, ratio of the mean of normalized spot volumes measured in GFP proteome compared to WT proteome;
pGFP, p value when comparing spot volumes measured in GFP proteome compared to WT proteome (only variations for which p<0.05 are considered);
GFP/WT, pattern of variation when comparing spot volumes measured in GFP proteome compared to WT proteome: C, constant; D, down-accumulated; U, up-accumulated;
Theo MW (Da), theoretical molecular weight;
Theo pI, theoretical isoelectric point.

Spot N°	Number of id per spot	Accession number	Protein name	Organism	Subcellular localization	Function	Functional categories	Mean HPPD / Mean WT	pHPPD	HPPD / WT	Mean GFP / Mean WT	pGFP	GFP / WT	Theo. MW	Theo. Pi
5	1	gi|47507010	Peptidylprolyl isomerase	*Nicotiana benthamiana*	Chloroplast	Protein folding	06. Protein destination and storage	8.33	0.015	U	33.33	0.00	U	22302	9.59
6	1	gi|47507010	Peptidylprolyl isomerase	*Nicotiana benthamiana*	Chloroplast	Protein folding	06. Protein destination and storage	4.00	0.03	U	14.28	0.02	U	22302	9.59
11	1	gi|76866940	Peroxiredoxin (thioredoxin peroxidase)	*Nicotiana tabacum*	Mitochondrion	Detoxification	11.06-Disease /Defence / Detoxification	1.45	0.04	U	3.7	0.02	U	33542	8.89
24	1	gi|21912927	Peroxiredoxin (thioredoxin peroxidase)	*Nicotiana tabacum*	Chloroplast	Detoxification	11.06-Disease /Defence / Detoxification	3.84	0.004	U	4.16	0.02	U	29818	8.2
25	1	gi|92011878	Peroxiredoxin (thioredoxin peroxidase)	*Nicotiana tabacum*	Chloroplast	Detoxification	11.06-Disease /Defence / Detoxification	2.86	0.006	U	3.7	0.01	U	31932	6.49
27	1	gi|19898	Photosystem II oxygen-evolving complex (23-kDa polypeptide)	*Nicotiana tabacum*	Chloroplast	Photosystem II component	02.30-Energy / Photosynthesis	0.31	0.01	D	0.48	0.04	D	27866	7.03
30	1	gi|19898	Photosystem II oxygen-evolving complex (23-kDa polypeptide)	*Nicotiana tabacum*	Chloroplast	Photosystem II component	02.30-Energy / Photosynthesis	0.89	0.88	C	8.33	0.01	U	27866	7.03
35	1	gi|22550386	Carbonic anhydrase	*Nicotiana tabacum*	Chloroplast	Calvin cycle	02.30-Energy / Photosynthesis	0.4	0.02	D	16.66	0,00	U	34431	6.19
36	1	gi|22550386	Carbonic anhydrase	*Nicotiana tabacum*	Chloroplast	Calvin cycle	02.30-Energy / Photosynthesis	0.61	0.04	D	1.31	0.45	C	34431	6.19
37	1	gi|22550386	Carbonic anhydrase	*Nicotiana tabacum*	Chloroplast	Calvin cycle	02.30-Energy / Photosynthesis	0.18	0.007	D	0.44	0.03	D	34431	6.19
42	1	gi|5381255	Peroxidase	*Nicotiana tabacum*	Chloroplast	Detoxification	11.06-Disease /Defence / Detoxification	20,00	0.008	U	1.37	0.71	C	35709	7.59
50	1	gi|11465970	Cytochrome f	*Nicotiana tabacum*	Chloroplast	Electron transfer (cytochrome *b6f* complex)	02.30-Energy / Photosynthesis	2.22	0.007	U	2.04	0.01	U	35224	9.12
52	1	gi|30013663	Ribulose-1.5-bisphosphate carboxylase (small subunit)	*Nicotiana tabacum*	Chloroplast	Calvin cycle	02.30-Energy / Photosynthesis	0.001	0.0003	D	0.08	0,00	D	20268	7.57
53	1	gi|30013663	Ribulose-1.5-bisphosphate carboxylase (small subunit)	*Nicotiana tabacum*	Chloroplast	Calvin cycle	02.30-Energy / Photosynthesis	0.75	0.04	D	0.08	0,00	D	20268	7.57
58	1	gi|22550386	Carbonic anhydrase	*Nicotiana tabacum*	Chloroplast	Calvin cycle	02.30-Energy / Photosynthesis	0.27	0.003	D	2,00	0.03	U	34431	6.19
60	1	gi|115473	Carbonic anhydrase	*Nicotiana tabacum*	Chloroplast	Calvin cycle	02.30-Energy / Photosynthesis	0.14	0.0005	D	0.23	0,00	D	34489	6.41
61	1	gi|22550386	Carbonic anhydrase	*Nicotiana tabacum*	Chloroplast	Calvin cycle	02.30-Energy / Photosynthesis	0.14	0.017	D	0.1	0.01	D	34431	6.19
68	1	gi|15225249	Glycine decarboxylase (P-protein)	*Arabidopsis thaliana*	Mitochondrion	Photorespiration	02.13-Energy / Respiration	0.63	0.002	D	1.33	0.04	C	113703	6.18
70	1	gi|559005	Ascorbate peroxidase	*Nicotiana tabacum*	Mitochondrion	Detoxification	11.06-Disease /Defence / Detoxification	0.36	0.04	D	4.76	0.03	U	27371	5.43
72	1	gi|92037383	SOUL heme-binding family protein	*Arabidopsis thaliana*	Chloroplast	Tetrapyrrole carrier protein	01.07-Metabolism / Cofactors	1.23	0.57	C	3.03	0.01	U	32673	7.74
73	1	gi|7331143	Chaperonin 21	*Lycopersicon esculentum*	Chloroplast	Protein folding	06. Protein destination and storage	1.89	0.013	U	1.25	0.22	C	26546	6.85
75	1	gi|11465949	Photosystem II protein D2	*Nicotiana tabacum*	Chloroplast	Photosystem II component	02.30-Energy / Photosynthesis	0.98	0.94	C	2.08	0.01	U	39510	5.33
76	1	gi|8347420	Cysteine protease	*Nicotiana tabacum*	Vacuolar	Proteolysis	06. Protein destination and storage	1.47	0.03	U	3.33	0,00	U	39175	6.95
78	1	gi|11134054	Photosystem II oxygen-evolving complex (33-kDa polypeptide)	*Nicotiana tabacum*	Chloroplast	Photosystem II component	02.30-Energy / Photosynthesis	0.42	0.023	D	0.39	0.02	D	35206	5.89
79	1	gi|147791852	Photosystem II oxygen-evolving complex (33-kDa polypeptide)	*Vitis vinifera*	Chloroplast	Photosystem II component	02.30-Energy / Photosynthesis	0.44	0.03	D	0.55	0.05	D	33213	5.87
80	1	gi|11134054	Photosystem II oxygen-evolving complex (33-kDa polypeptide)	*Nicotiana tabacum*	Chloroplast	Photosystem II component	02.30-Energy / Photosynthesis	0.42	0.03	D	0.47	0.04	D	35367	5.84
84	1	gi|13310811	Ankyrin-repeat protein HBP1	*Nicotiana tabacum*	Cytosol	Plasmodesmal gating	06. Protein destination and storage	0.05	0.00055	D	0.3	0,00	D	37294	4.45
87	1	gi|115448091	Phosphoribulokinase	*Oryza sativ*	Chloroplast	Calvin cycle	02.30-Energy / Photosynthesis	0.13	0.02	D	0.625	0.04	D	44837	5.68
88	1	gi|125578	Phosphoribulokinase	*Mesembryanthemum crystallinum*	Chloroplast	Calvin cycle	02.30-Energy / Photosynthesis	0.19	0.05	D	0.507	0.02	D	44086	6.03

92	1	gi|4827253	Fructose-bisphosphate aldolase	*Nicotiana paniculata*	Chloroplast	Calvin cycle	02.30-Energy / Photosynthesis	4,00	0.007	U	1.51	0.41	C	42789	6.38
99	1	gi|32746733	Chloroplast mRNA-binding protein	*Nicotiana tabacum*	Chloroplast	Protein synthesis	05.04-Protein synthesis / Translation factors	1.31	0.41	C	2.12	0.01	U	44217	6.51
100	1	gi|147773479	Chloroplast mRNA-binding protein	*Vitis vinifera*	Chloroplast	Protein synthesis	05.04-Protein synthesis / Translation factors	1.85	0.02	U	1.43	0.04	U	42304	8.74
104	1	gi|120661	Glyceraldehyde-3-phosphate dehydrogenase	*Nicotiana tabacum*	Chloroplast	Calvin cycle	02.30-Energy / Photosynthesis	1.56	0.23	C	3.03	0.01	U	41837	6.6
109	1	gi|1707878	Glycine decarboxylase (T-protein)	*Solanum tuberosum*	Mitochondrion	Photorespiration	02.13-Energy / Respiration	0.41	0.000001	D	1.05	0.67	C	44249	8.77
110	1	gi|1707878	Glycine decarboxylase (T-protein)	*Solanum tuberosum*	Mitochondrion	Photorespiration	02.13-Energy / Respiration	0.44	0.006	D	0.38	0,00	D	44249	8.77
113	1	gi|120665	Glyceraldehyde-3-phosphate dehydrogenase	*Nicotiana tabacum*	Chloroplast	Calvin cycle	02.30-Energy / Photosynthesis	0.32	0.01	D	0.39	0.02	D	47440	8.83
115	1	gi|120665	Glyceraldehyde-3-phosphate dehydrogenase	*Nicotiana tabacum*	Chloroplast	Calvin cycle	02.30-Energy / Photosynthesis	0.38	0.004	D	1.07	0.75	C	47440	8.83
123	1	gi|2499497	Phosphoglycerate kinase	*Nicotiana tabacum*	Chloroplast	Calvin cycle	02.30-Energy / Photosynthesis	0.57	0.01	D	0.49	0.04	D	50146	8.48
124	1	gi|218312	Translation elongation factor TuB	*Nicotiana sylvestris*	Chloroplast	Protein synthesis	05.04-Protein synthesis / Translation factors	0.54	0.03	D	0.78	0.05	C	46672	5.7
125	1	gi|68566313	Translation elongation factor TuA	*Nicotiana tabacum*	Chloroplast	Protein synthesis	05.04-Protein synthesis / Translation factors	0.6	0.04	D	0.66	0.05	D	51924	6.34
132	1	gi|6683504	Actin	*Mimosa pudica*	Cytosol	Cytoskeleton	09.04-Cell structure / Cytoskeleton	0.19	0.002	D	1.11	0.55	C	41703	5.31
134	1	gi|10720247	RuBisCO activase	*Solanum pennellii*	Chloroplast	Calvin cycle	02.30-Energy / Photosynthesis	2.56	0.05	U	3.125	0.04	U	50669	8.61
139	1	gi|30025966	Heat shock protein (HSP 70)	*Nicotiana tabacum*	Cytosol	Protein folding	06. Protein destination and storage	0.42	0.003	D	0.86	0.14	C	70832	5.17
156	1	gi|4262869	Ribulose-1.5-bisphosphate carboxylase (large subunit)	*Nicotiana tabacum*	Chloroplast	Calvin cycle	02.30-Energy / Photosynthesis	0.27	0.02	D	0.11	0.02	D	52895	6.29
158	1	gi|132000	Ribulose-1.5-bisphosphate carboxylase (large subunit)	*Nicotiana acuminata*	Chloroplast	Calvin cycle	02.30-Energy / Photosynthesis	0.38	0.03	D	0.39	0.03	D	52908	6.41
161	1	gi|462187	Serine hydroxymethyltransferase	*Pisum sativum*	Mitochondrion	Photorespiration	02.13-Energy / Respiration	2.5	0.012	U	0.97	0.93	C	57256	8.71
